# Extranodal Rosai-Dorfman Disease of Breast Mimicker of Breast Malignancy

**DOI:** 10.1055/s-0043-1760763

**Published:** 2024-04-11

**Authors:** Pokhraj Prakashchandra Suthar, Adithya Sivakumar, Gladson Scaria, Jagadeesh S. Singh

**Affiliations:** 1 Department of Radiology and Nuclear Medicine, Rush University Medical Center, Chicago, Illinois, United States; 2 Department of Pathology, Rush University Medical Center, Chicago, Illinois, United States

**Keywords:** ^18^ F-FDG PET/CT, breast, histopathology, mammogram, Rosai-Dorfman disease, ultrasound

## Abstract

Rosai-Dorfman-Destombes (RDD) disease is also known as sinus histiocytosis with massive lymphadenopathy. It is an uncommon heterogeneous disease of children and young adults. Most of the patients with RDD generally present with painless lymphadenopathy, while extranodal and multisystem manifestation of the disease is unusual. The diagnosis is based on the imaging with clinicopathological correlation. Flourine-18 fluorodeoxyglucose positron emission tomography/computed tomography is useful for the initial staging of the RDD lesions, which have similar appearance and avidity like intermediate and high-grade lymphomas. Here, we present the case of a 55-year-old female presented with left breast mass that turned out to be the extranodal Rosai-Dorfman disease.

## Introduction

The Rosai-Dorfman-Destombes (RDD) is sinus histiocytosis and is common in younger age group. Nodal form of the disease is present with massive painless lymphadenopathy, while extranodal and multisystem involvement is uncommon. The multisystem manifestations are present in up to 19% of cases. 1 Clinical features of RDD overlap with the Hodgkin's or non-Hodgkin's lymphoma. Extranodal disease is mostly seen in the cutaneous planes, bone, central nervous systems, with breast involvement being uncommon. Despite its usually benign course, extranodal RDD may present as hypermetabolic breast lesion on flourine-18 fluorodeoxyglucose positron emission tomography/computed tomography ( ^18^ F-FDG PET/CT) and mimic breast malignancy or lymphoma.

## Case History

A 55-year-old female with a history of hypertension, hyperlipidemia, asthma, and type-2 diabetes mellitus presented with a new left breast palpable mass for 2 weeks and enlarging right upper abdominal mass lesion. The patient had associated complaints of fatigue and lost 5 lbs of weight. She denied a history of trauma, fever, night sweats, chest pain, shortness of breath, and abdominal symptoms. The patient had a history of a right upper quadrant abdominal lump, which was more prominent on bending forward. On examination, the left breast and upper abdominal lesions were nonerythematous and nontender. The vital signs and the rest of the examinations were within normal limits. The patient had impaired diabetes control with hemoglobin A1C of 11.9% (reference range < 5.6%). The erythrocyte sedimentation rate was elevated (46 mm/hour by the Westergren method; reference range: 0–30 mm/hour). The white blood cell count was within the normal range (6.69 K/µL; reference range: 4–10 K/µL), but the differential lymphocyte percentage was elevated (48.3%; reference range: 20–45%). The rest of the laboratory workup was within normal limits.

**Fig. 1:**
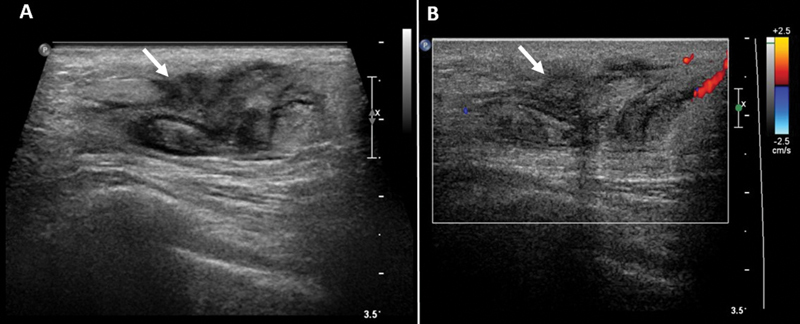
Left breast ultrasound ( **A** ) gray scale and ( **B** ) color duplex demonstrate an ill-defined 3.3 × 1.2 × 2.9 cm size heterogeneously hypoechoic mass lesion at 4 o'clock position 14 cm from the nipple in left breast with minimal marginal vascularity ( *white arrow* in **A** and **B** ).

**Fig. 2:**
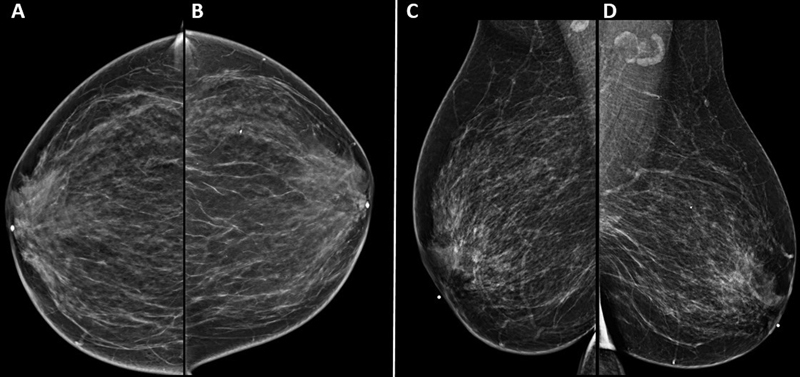
Bilateral breast mammogram ( **A** ) right craniocaudal (CC), ( **B** ) left CC, ( **C** ) right mediolateral oblique (MLO), and ( **D** ) left MLO views. Heterogeneously dense bilateral breast parenchyma with no apparent dominant mass, suspicious calcification, or areas of architectural distortion identified. No abnormal axillary or subpectoral lymphadenopathy.

**Fig. 3:**
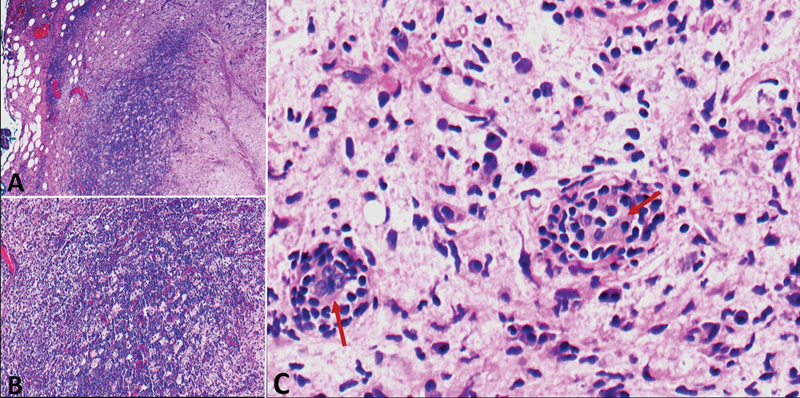
Histopathology examination of ultrasound-guided core-needle left breast biopsy hematoxylin and eosin stain ( **A** ) 4× magnification, ( **B** ) 10× magnification, and ( **C** ) 50× magnification. ( **A** , **B** ) An infiltration of lesional histocytes with associated fibrosis and inflammatory cells composed of lymphocyte and plasma cells. ( **C** ) Emperipolesis with lesioned histocytes engulfing inflammatory cells associated with fibrosis and inflammatory cells ( *maroon arrows* ).

**Fig. 4:**
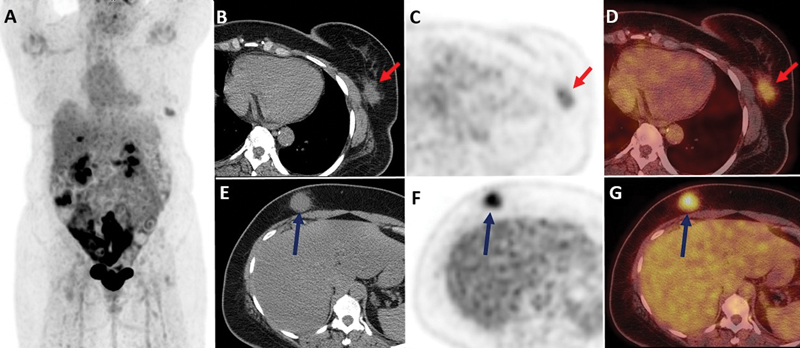
Fluorine 18-fluorodeoxyglucose positron emission tomography/computed tomography (PET/CT) image: ( **A** ) Maximum intensity projection, ( **B** , **E** ) unenhanced, ( **C** , **F** ), axial PET, and ( **D** , **G** ) transaxial fused PET/CT images. Hypermetabolic soft tissue density lesion in the lower outer part of the left breast without infiltration of left pectoralis muscles ( *red arrows* in **B** – **D** ). Additionally, hypermetabolic soft tissue density lesion in the right upper quadrant in subcutaneous planes ( *dark blue arrows* in **B** – **D** ).

The patient underwent left breast ultrasound with a mammogram that revealed an ill-defined 3.3 × 1.2 × 2.9 cm size heterogeneously hypoechoic mass lesion at 4 o'clock position 14 cm from the nipple ( Fig. 1 ). On mammography, the breasts were heterogeneously dense with no apparent dominant mass, suspicious calcification, or areas of architectural distortion identified. No abnormal axillary or subpectoral lymphadenopathy was found ( Fig. 2 ). She underwent an ultrasound-guided core needle left breast biopsy that showed emperipolesis with lesioned histocytes engulfing inflammatory cells with associated fibrosis and inflammatory cells ( Fig. 3 ). Based on clinical, laboratory, and imaging findings, diagnosis of an extranodal Rosai-Dorfman disease was postulated. As a part of the disease, surveillance to know the extent of the disease ^18^ F-FDG PET/CT was performed. There was a hypermetabolic soft tissue density lesion in the lower outer part of the left breast without infiltration of left pectoralis muscles and no axillary lymphadenopathy. Additionally, hypermetabolic soft tissue density lesion was found in the right upper quadrant in subcutaneous planes with few subcentimeters minimally FDG avid nodules adjacent to it ( Fig. 4 ). Surgical excision of the left breast and anterior abdominal wall lesions was performed with an uneventful postoperative course. Currently, the patient is on disease surveillance without any symptoms.

## Discussion

RDD disease is a rare disorder that classically presents with massive bilateral, painless lymphadenopathy that can be associated with fever, weight loss, and night sweats. It is described as a histiocytic disorder that can be classified in several different ways, including the classic nodal, extranodal, neoplasia-associated, and immune disease-associated forms. 1 Extranodal manifestations are present in up to 43% of cases, with multisystem involvement noted in 19% of cases. Other manifestations include cutaneous, central nervous system, bone, intrathoracic, and gastrointestinal involvement. Due to their nonspecific symptoms and presentation, the disease can mimic other pathologies, including Hodgkin's and non-Hodgkin's lymphoma, Langerhans cell histiocytosis, and reactive lymphadenopathy.

The disease has a prevalence of 1:200,000 and seen more frequently in children and young adults, with the mean patient age of 20.6 years. An initial study showed that those affected are predominantly male and of African descent. Extranodal disease was found to be more frequent in elderly patients. 2 On laboratory evaluation, there are many neutrophil cells on complete blood count, polyclonal hypergammaglobulinemia on serum immunoglobin testing, and a high erythrocyte sedimentation rate. 3

For children, chest X-ray with neck and abdominal ultrasounds are recommended. Older patients begin their evaluation with a CT of the neck/chest/abdomen and pelvis. In the breast, mammography may demonstrate a high-density mass. 4 In contrast, our patient was noted to have focal asymmetry on initial mammography screening. For those with suspected extranodal involvement, FDG-PET/CT can be used for initial staging. RDD lesions have the appearance and metabolic activity similar to intermediate and high-grade lymphomas, which can be differential diagnostic considerations. Enlarged lymph nodes reveal homogenous enhancement on CT images and hypermetabolism on FDG-PET. FDG-PET has also been used as an imaging study to check the response of the disease to treatment. 5

In one review, it was found that a median of two biopsies was required to establish a diagnosis. Pathology can show expression of CD163, CD68, and S100 antigens, along with enlarged histiocytes. In extranodal lesions, however, these histiocytes were not frequently found, usually obscured with inflammatory background. 6

The disease spontaneously revolves in approximately of 20 to 50% of patients, but it can lead to organ dysfunction due to nodal enlargement. Treatment options vary depending on the patient. The most common option is surgical excision of the involved sites. Other options include corticosteroids, radiation therapy, rituximab, azathioprine, or 6-mercaptopurine. The mortality rate is reported as 7%. 7

## Conclusion

RDD disease is a sinus histiocytosis and commonly present with nodal disease. The extranodal and multisystem manifestation of the disease is uncommon. ^18^ F-FDG PET/CT is useful for the initial staging and to check for the disease burden. Breast involvement in RDD may present as hypermetabolic masses on PET/CT and may be confused with malignancy or lymphoma. The diagnosis is based on combining imaging and clinicopathological findings.
